# Improving Internal Medicine Residents’ Colorectal Cancer Screening Knowledge Using a Smartphone App: Pilot Study

**DOI:** 10.2196/mededu.9635

**Published:** 2018-03-13

**Authors:** Zubair Khan, Umar Darr, Muhammad Ali Khan, Mohamad Nawras, Basmah Khalil, Yousef Abdel-Aziz, Yaseen Alastal, William Barnett, Thomas Sodeman, Ali Nawras

**Affiliations:** ^1^ Department of Internal Medicine University of Toledo Medical Center Toledo, OH United States; ^2^ Department of Gastroenterology University of Tennessee Health Science Center Memphis, TN United States; ^3^ Department of Gastroenterology University of Toledo Medical Center Toledo, OH United States

**Keywords:** colorectal cancer, cancer screening, early detection of cancer, residents’ education, smartphone, mobile apps

## Abstract

**Background:**

Colorectal cancer (CRC) is the third most common type of cancer and the second leading cause of cancer death in the United States. About one in three adults in the United States is not getting the CRC screening as recommended. Internal medicine residents are deficient in CRC screening knowledge.

**Objective:**

The objective of our study was to assess the improvement in internal medicine residents’ CRC screening knowledge via a pilot approach using a smartphone app.

**Methods:**

We designed a questionnaire based on the CRC screening guidelines of the American Cancer Society, American College of Gastroenterology, and US Preventive Services Task Force. We emailed the questionnaire via a SurveyMonkey link to all the residents of an internal medicine department to assess their knowledge of CRC screening guidelines. Then we designed an educational intervention in the form of a smartphone app containing all the knowledge about the CRC screening guidelines. The residents were introduced to the app and asked to download it onto their smartphones. We repeated the survey to test for changes in the residents’ knowledge after publication of the smartphone app and compared the responses with the previous survey. We applied the Pearson chi-square test and the Fisher exact test to look for statistical significance.

**Results:**

A total of 50 residents completed the first survey and 41 completed the second survey after publication of the app. Areas of CRC screening that showed statistically significant improvement (*P*<.05) were age at which CRC screening was started in African Americans, preventive tests being ordered first, identification of CRC screening tests, identification of preventive and detection methods, following up positive tests with colonoscopy, follow-up after colonoscopy findings, and CRC surveillance in diseases.

**Conclusions:**

In this modern era of smartphones and gadgets, developing a smartphone-based app or educational tool is a novel idea and can help improve residents’ knowledge about CRC screening.

## Introduction

Colorectal cancer (CRC) is the third most commonly diagnosed cancer among both men and women in the United States. It is the second leading cause of cancer-related death overall. Incidence and mortality rates have been declining because of increased awareness of risk factors such as smoking and red meat consumption, and improvement in screening rates and treatment modalities [1-3]. According to the American Cancer Society (ACS), 135,430 new cases and 50,260 deaths from CRC were expected to occur in 2017, and the lifetime risk of developing CRC is about 1 in 21 (4.7%) for men and 1 in 23 (4.4%) for women [4]. The management of CRC is associated with substantial health care costs, with national expenditures exceeding US $14 billion annually [5,6]. There are striking disparities by age, race, and tumor subsite despite a reduction in CRC incidence and mortality overall. The goal of further reducing CRC incidence and mortality can be achieved by ensuring access to high-quality health care, incentivizing healthy lifestyles, and increasing CRC screening. Meester and colleagues and others estimated that achieving the US National Colorectal Cancer Roundtable’s goal of increasing screening prevalence to 80% by 2018 would prevent 277,000 CRC cases and 203,000 deaths by 2030 [7-10]. About one in three adults in the United States is not getting CRC screening as recommended. According to the US National Health Interview Survey, CRC screening in accordance with the guidelines among adults 50 years of age and older increased from 34% in 2000 to 63% in 2015 [11].

Generally, it is expected that as resident physicians advance in their training, CRC screening rates should improve with the expected improvement in knowledge of CRC screening. Wong measured performance outcomes in multiple screening categories over 3 years of training and found that actual patient screening rates were similar across all years [12]. One of the reasons for this lack of improvement in CRC screening could be residents’ deficient knowledge about CRC screening, even though guidelines from the American College of Gastroenterology (ACG), ACS, and US Preventive Services Task Force (USPSTF) are mostly in agreement about screening modalities and age [13-15]. Akerman et al [16] assessed residents’ CRC screening knowledge via a Web-based survey and concluded that there were many deficiencies. They concluded that fecal occult blood testing for screening purposes remains undervalued, and confusion about administering the test persists. The distinction between screening and prevention needs further reinforcement [16].

Primary medical care of many underserved populations is dependent on resident outpatient practices. The physician-in-training role in health maintenance and screening has been assessed by various studies [17-21]. Other factors could be responsible for the compromise in effective health maintenance and screening in resident practice in addition to residents’ knowledge deficiencies. One of these factors could be provider turnover every few years. Some studies even estimate that as many as 50% of patients are lost to follow-up of their chronic medical conditions and screenings when resident physicians graduate and pass their patients on to new providers [22].

Besides addressing other factors to improve health maintenance and screening in residents’ practice, improving medical knowledge about preventive health and screening is the key. One of the reasons for residents’ deficient knowledge about CRC screening is lack of training and educational tools. We conducted this comprehensive study to improve internal medicine residents’ CRC screening knowledge via a pilot approach using a smartphone app.

## Methods

### Survey Design

This pilot study was completed in 3 parts. Initially, we designed a questionnaire based on the CRC screening guidelines of the ACS, ACG, and USPSTF; we then requested institutional review board approval. The institutional review board of the University of Toledo Medical Center then granted the request for approval after reviewing the app and the survey questionnaire (no. 201713). The survey contained 14 questions on 7 areas of CRC screening, Textbox 1 outlines. We emailed the questionnaire via a SurveyMonkey (SurveyMonkey Inc, San Mateo, CA) link to all the residents of an internal medicine department. We analyzed the responses after 4 weeks. The first question simply asked for the year of training, to create a subset for analysis by year of training. Respondents had the ability to answer with multiple correct choices for some questions, reflecting the multiple options presented in the source guidelines. Multimedia Appendix 1 shows the survey form.

**The 7 areas of colorectal cancer screening tested in the survey and covered in the app.**

Screening in average risk and with positive family history of colorectal cancer

Identification of screening tests

Prevention methods

Detection methods

Following up positive tests with colonoscopy

Follow-up after colonoscopy findings

Surveillance in diseases

### App Design

**Figure 1:**
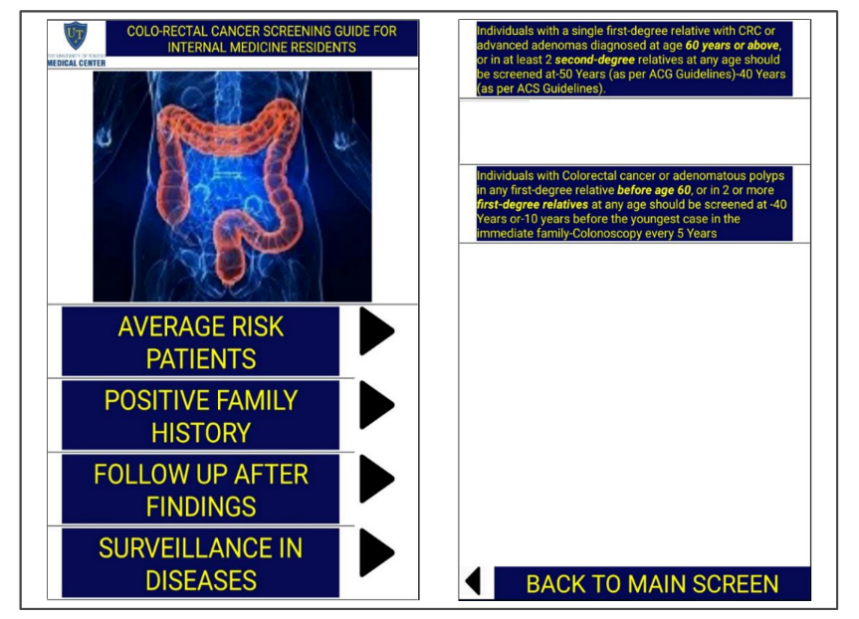
Screenshots of the main screen and family history screen. ACS: American Cancer Society; ACG: American College of Gastroenterology; CRC: colorectal cancer.

**Figure 2:**
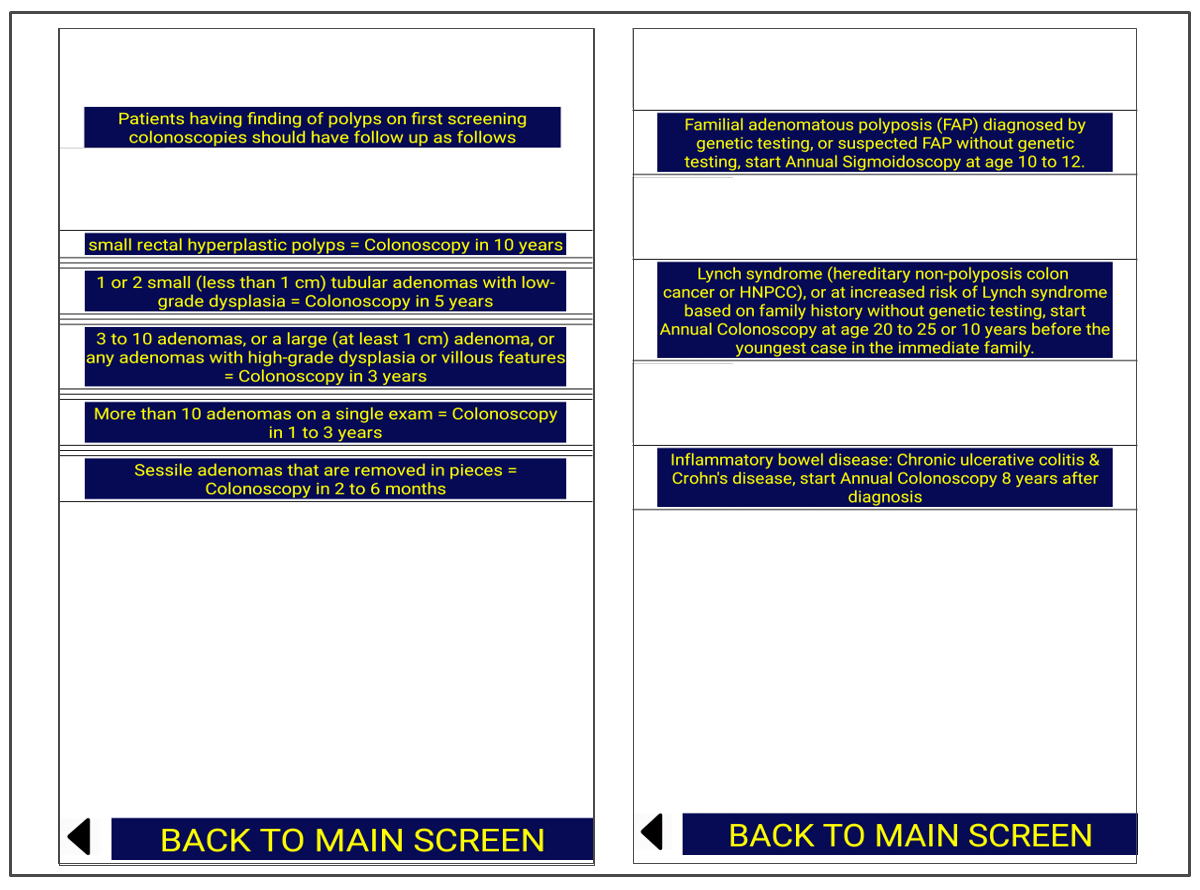
Screens showing follow-up after findings and surveillance in diseases.

**Figure 3:**
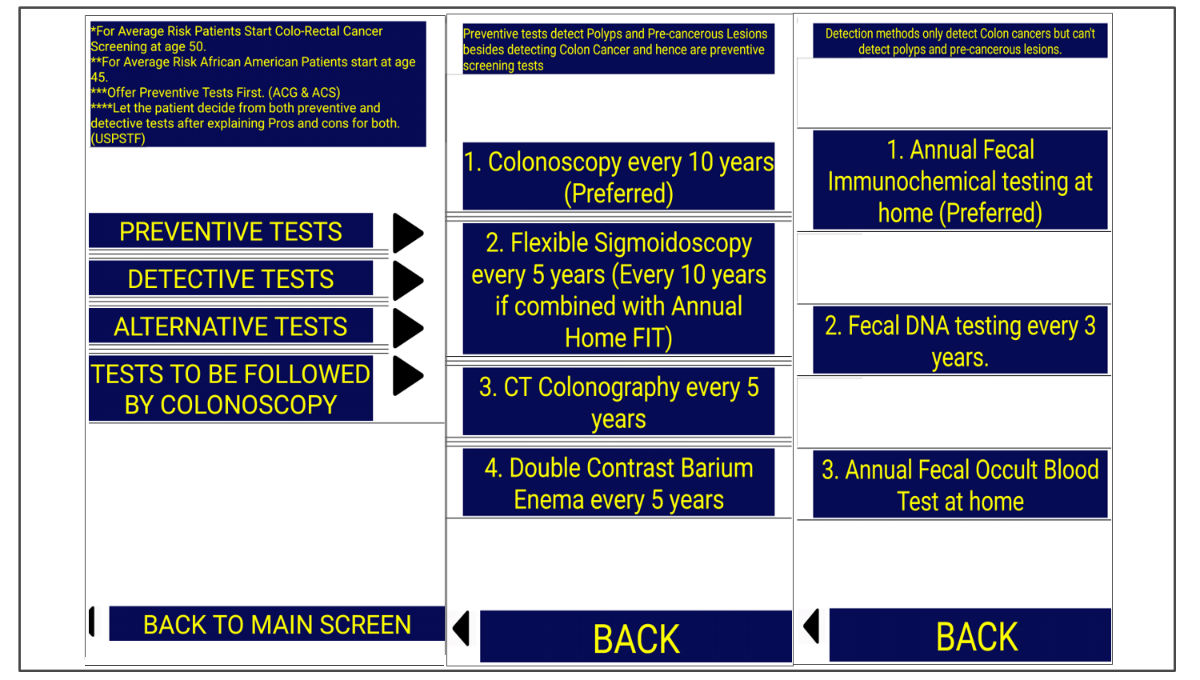
Screens giving information about preventive and detection tests in average-risk patients. ACS: American Cancer Society; ACG: American College of Gastroenterology; FIT: fecal immunochemical test; USPSTF: US Preventive Services Task Force.

**Figure 4:**
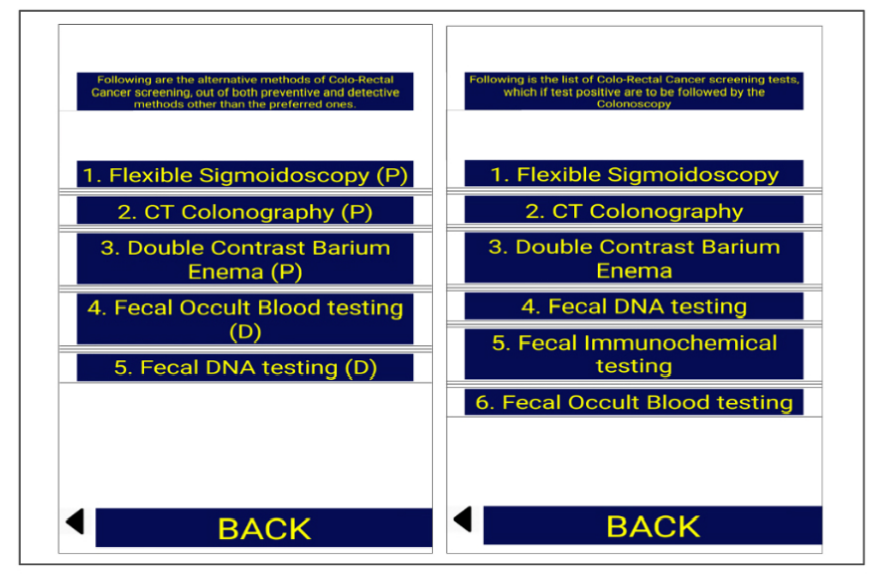
Screens showing alternative methods of colon cancer screening and screening tests to be followed by colonoscopy if positive. CT: computed tomography.

In the second part, we designed a smartphone app. The decision to use a smartphone app for education was purely experimental and was based on the recent advancement in technology of smartphones and gadgets and the subsequent growth of the smartphone app industry. First, we collected information about CRC screening based on the ACG, ACS, and USPSTF guidelines, and then made a screen tree based on this information. The screen tree consisted of a total of 9 screens, including the main screen, as Figures 1,2,3, and 4 show. The smartphone app was created on an online app creation portal (Mobincube, San Francisco, CA, USA). We designed the app keeping in mind simplicity yet ensuring good visibility of the information. The portal subscription we obtained for the app creation and publication was without advertisements to avoid any conflicts of interest. We tested the trial version of the app on a smartphone and a tablet before publication.

Then we uploaded the app onto the Google Developers Console (Google Inc, Mountain View, CA, USA) and published it the Google Play Store (Google Inc) for Android users. We also sent the app to Apple support for testing before publication in the App Store (Apple Inc, Cupertino, CA, USA). All the residents were introduced to the app via emails and flyers. We tracked the number of app downloads via a developer account in both the Play Store and the App Store. The app was purely educational and there were no interactive components in the app. The purpose of the app was to improve residents’ knowledge about CRC screening.

In the third and final part, we repeated the survey after 4 weeks and compared the responses with those of the first survey. Weekly reminders were sent to residents to complete both the surveys. We gave residents no incentives to complete the surveys. We applied the Pearson chi-square test and the Fisher exact test to look for statistical significance.

## Results

### Survey Response Rates

We analyzed and compared the data in 3 subsets. In the first subset, we compared responses to the survey questions from respondents in the same training year to determine improvement in knowledge in each training year individually after publication of the smartphone app. In the second subset analysis, we compared responses between each of the 3 resident training years (ie, postgraduate year [PGY]-1, PGY-2, and PGY-3) to look for differences in knowledge between different training levels at baseline and differences in knowledge improvement after publication of the smartphone app. In the third and final analysis, we analyzed responses in aggregate to look for overall improvement in knowledge.

We emailed the survey link to 59 residents during the first phase and allowed 4 weeks for completion of the survey, with weekly reminders sent via email. A total of 50 residents completed the survey, for response rate of 85%. Of the 50 respondents, there were 22 PGY-1 residents, 15 PGY-2 residents, and 13 PGY-3 residents. After publication of the app, we emailed the survey link again. A total of 41 residents responded to the second survey, for a response rate of 69%; of the respondents, there were 20 PGY-1 residents, 11 PGY-2 residents, and 10 PGY-3 residents.

### Assessment of Residents’ Knowledge

The first few survey questions assessed the resident’s knowledge about CRC screening in average-risk patients and with a family history of CRC (Multimedia Appendix 2). Most of the residents correctly identified the screening age in such patients. The residents were lacking knowledge about the ACG recommendation to start screening for CRC in African Americans at age 45 years. But after using the smartphone app, their knowledge improved significantly, from only 4 residents (8%) responding correctly before using the app to 29 residents (71%) responding correctly after using the app (*P*<.001). Although not statistically significant, knowledge about offering preventive tests first (*P*=.01) and offering colonoscopy every 5 years to patients with family history of CRC in a first-degree relative at age less than 60 years (*P*=.17) all improved.

When asked to identify screening modalities for CRC screening, many residents were lacking the knowledge about the various tests that can be offered. The number of correct responses indicating their knowledge about computed tomography (CT) colonography every 5 years, double-contrast barium enema every 5 years, sigmoidoscopy every 10 years with annual fecal immunochemical testing at home, and fecal DNA testing every 1 to 3 years increased with statistical significance after intervention (Multimedia Appendix 3). The residents were also tested on their ability to identify preventive tests, which can detect precancerous polypoid lesions. Most residents could identify only colonoscopy every 10 years as a preventive test at baseline, but after the intervention, more of them, at all training levels, correctly identified flexible sigmoidoscopy every 5 years, CT colonography every 5 years, and double-contrast barium enema every 5 years as preventive tests (Multimedia Appendix 4).

Detection methods only can detect CRC; they can’t prevent it as prevention methods do by detecting precancerous polypoid lesions. Stool-based CRC screening tests are the detection methods. Residents knowledge for correctly identifying detection methods was not satisfactory at baseline but improved significantly after education with the app, and they were able to identify annual fecal immunochemical testing, fecal occult blood testing at home, and fecal DNA testing every 1 to 3 years as CRC detection methods (Multimedia Appendix 5). Most residents could identify sigmoidoscopy and fecal occult blood testing as the tests that are to be followed by colonoscopy if the test result is positive. At baseline, they did not all know that positive results on CT colonography, double-contrast barium enema, fecal immunochemical testing, and fecal DNA testing should also be followed by colonoscopy. After using the smartphone app, however, more of the residents identified these tests as needing to be followed by colonoscopy (Multimedia Appendix 6).

The residents were also tested on the recommended follow-up after colonoscopy screening. Overall, the residents’ knowledge was not satisfactory on the follow-up periods of 10 years in the case of small hyperplastic rectal polyps being found, 5 years for 1 or 2 small tubular adenomas, 3 years for 3 to 10 adenomas, 1 to 3 years for more than 10 adenomas, and 2 to 6 months for sessile adenomas. Knowledge improved, but this was statistically significant only in the case of knowledge about the follow-up of sessile adenomas and of more than 10 adenomas (Multimedia Appendix 7). Finally, residents were asked about surveillance for CRC in familial adenomatous polyposis, Lynch syndrome, and inflammatory bowel disease. Residents’ knowledge about CRC surveillance in familial adenomatous polyposis was lacking before using the smartphone app but improved significantly after the educational intervention (Multimedia Appendix 8).

## Discussion

### Principal Findings

Most of the recommendations for CRC screening from the ACG, ACS, and USPSTF are similar. For our study, we extracted commonalities and only tested recommendations that were similar between all of these guidelines. Apart from these organizations, others also publish guidelines, most of which reinforce the already-stated recommendations, but they also make some new recommendations, adding to the confusion for residents and other health care professionals. Recently, the US Multi-Society Task Force on Colorectal Cancer made new recommendations, in which they divided the screening tests into three tiers based on performance features, costs, and practical considerations [23]. In our study, we tested the knowledge of residents to identify these screening tests but didn’t test for division of these tests into three tiers.

Our study showed that, regardless of the levels of training, residents in one internal medicine department were lacking knowledge about CRC screening. This finding agrees with that of Sharma et al [24,25], who investigated the understanding of CRC screening among primary care physicians and internists. There was no difference in residents’ knowledge between baseline and after the educational intervention in the form of a smartphone app: knowledge improved equally among all 3 PGY levels. This finding is consistent with a study in 2005 [26], which showed no statistically significant difference in CRC screening rates between different years of training.

Our study was different from previously reported ones in that, first, ours was very comprehensive and covered all aspects of CRC screening and, second, it used the novel approach of a smartphone app for education of the residents. Previously reported studies, such as that of Akerman et al [16], only tested for identification of CRC screening tests. Beyond testing for knowledge of CRC screening in average-risk patients and with a family history of CRC, we also tested the important concept of differentiation between prevention and detection tests, which is acknowledged in the ACG and ACS guidelines. Residents were lacking knowledge in other areas of CRC screening, such as identifying tests other than colonoscopy, screening in African American patients, following up positive tests with colonoscopy, follow-up after colonoscopy findings, and surveillance for CRC in various diseases such as familial adenomatous polyposis, Lynch syndrome, and inflammatory bowel disease.

Our study showed that residents were knowledgeable about screening of average-risk patients with colonoscopy at age 50 years and those with a family history of CRC. Because ACS guidelines state that colonoscopy should be offered at age 40 years to patients with a family history of CRC in a first-degree relative at or over age 60 years and ACG guidelines state that screening in these patients should be the same as for average-risk patients, we considered both responses correct in our survey analysis. The areas that showed improved knowledge after use of the smartphone app were correctly identifying all of the CRC screening tests, differentiating between prevention and detection tests, and correctly identifying these tests. Also, the postsurvey results showed improved knowledge of screening tests that need to be followed by colonoscopy if the test result is positive. The areas that didn’t improve much were the follow-up of a screening colonoscopy in case of findings, as well as surveillance for CRC in various diseases.

Residents’ knowledge about alternative CRC screening methods is important, as some patients do not wish to have colonoscopies because of the invasive nature of the test. Residents can only offer alternative, less-invasive methods if they have knowledge about them. Although the use of screening colonoscopies has increased over the last few years, it is still far from the National Colorectal Cancer Roundtables’ goal of achieving 80% by 2018, and awareness of alternative methods is important in achieving this target. It is important for residents to know about follow-up after colonoscopy findings and surveillance for CRC in various diseases, as residents ultimately take care of the primary needs of these patients in the clinic.

Various educational strategies have been employed in the past for improving knowledge of CRC screening, ranging from didactic lectures, as shown by Lane et al [27], to an interactive case-based model, as shown by Schroy et al [28]. These educational methods showed variable improvement in knowledge, and additional interventions may be needed to improve screening performance. In this modern era of smartphones and gadgets, developing a smartphone-based app or educational tool can work very well for residents’ education, as shown by Shaw and Tan [29]. Using a smartphone app to improve internal medicine residents’ knowledge of CRC screening is a novel idea and worked very well in our study.

### Study Limitations

Our response rate was good, but our study was limited by being a single-center study with a small sample size. This intervention can be expanded to other institutions to determine the validity of our results in a multicenter setting. In addition, this study did not check whether the residents’ improved knowledge translated into their clinical practice in terms of an improved CRC screening rate. A multi-institutional study is being planned, and the original study will be expanded to determine the app’s effect on the screening rate. Here it is important to mention that, although this study was not intended to observe a change in practice, a few residents reported that the app was a readily available tool on their smartphone and was helpful to them when they encountered the issue of CRC screening of their patients in an outpatient setting.

### Conclusion

While residents seem knowledgeable about colonoscopic CRC screening in average-risk patients, we found significant deficiencies in other areas of the comprehensive evaluation. A smartphone-based app or educational tool is a novel idea and can help improve residents’ knowledge about CRC screening. A smartphone-based educational tool can be a part of residents’ orientation before the start of their residency to reinforce their knowledge about age-appropriate and specific screenings.

## Appendix

Multimedia Appendix 1 Survey form emailed via SurveyMonkey to residents.

Multimedia Appendix 2 Screening in average-risk patients and with a positive family history of colorectal cancer.

Multimedia Appendix 3 Identification of colorectal cancer screening tests.

Multimedia Appendix 4 Identification of colorectal cancer prevention methods.

Multimedia Appendix 5 Identification of colorectal cancer detection methods.

Multimedia Appendix 6 Identification of positive tests to be followed by colonoscopy.

Multimedia Appendix 7 Identification of follow-up after colonoscopy.

Multimedia Appendix 8 Identification of surveillance for colorectal cancer in various diseases.

## Abbreviations

ACS: American Cancer Society
ACG: American College of Gastroenterology
CRC: colorectal cancer
CT: computed tomography
FIT: fecal immunochemical test
PGY: postgraduate year
USPSTF: US Preventive Services Task Force
