# Supplementary material for: Improving Internal Medicine Residents’ Colorectal Cancer Screening Knowledge Using a Smartphone App: Pilot Study
Source: JMIR Med Educ. 2018 Mar 13;4(1):e10. doi: 10.2196/mededu.9635 (PMC5871737; doi:10.2196/mededu.9635)
Supplement: Multimedia Appendix 1 [file mededu_v4i1e10_app1.pdf]

Colo-Rectal Cancer Screening Survey

I am Zubair Khan MD. from the University of Toledo Internal Medicine Residency.

I am conducting a survey with my colleagues on the “Internal Medicine Residents Knowledge of Colorectal Cancer Screening before and after Educational Strategies (including development of a smart phone application) as assessed by a web based survey”. The survey will help us to evaluate residents’ knowledge about colo-rectal cancer screening and to recommend the educational strategies needed for the improvement.

We are inviting you to participate in a survey regarding your knowledge about colo-rectal cancer screening, this survey should take approximately 10 minutes. Your participation is voluntary, and the survey is not part of any course grade. If you do not wish to participate, you may stop at any time. Responses will be completely anonymous. There are minimal risks associated with this survey, such as the stress of completing survey. Taking part in this survey is your agreement to participate.

If you have any questions regarding the survey, contact Zubair Khan MD, Internal Medicine Residency, zubair.khan@utoledo.edu

[UT IRB# 201713]

- I agree to Participate in the survey.
- I don't want to participate in the survey.

2. What is your PGY level?

PGY 1

PGY 2

PGY 3

3. For average risk patients at what age you start Colorectal Cancer screening ?

40 Years

45 Years

50 Years

55 Years

4. For Average Risk African American patients at what age you start colorectal

cancer screening ?

40 Years

45 Years

50 Years

55 Years

5. Which of the following should be offered first to the patient who is due for CRC

screening w

Preventive tests

Detection tests

6. Individuals with a single first-degree relative with CRC or advanced adenomas

diagnosed at age >=60 years, or in at least 2 second-degree relatives at any age should be screened at

40 Years

45 Years

50 Years

55 Years

7. Individuals with Colorectal cancer or adenomatous polyps in any first-degree relative before age 60, or in 2 or more first-degree relatives at any age should be screened at

40 Years

45 Years

50 Years

55 Years

8. Screening test of choice for patients with first degree relatives with CRC before age 60 is

Colonoscopy every 10 years starting at age 40

Flexible sigmoidoscopy every 5 years starting at age 50

FOBT annually at office at age 40

CT Colonography every 5 years starting at age 50

Fecal immunochemical testing annually at home starting at age 40

Double contrast barium enema every 5 years starting at age 50

Colonoscopy every 5 years starting at age 40

9. Which of the following tests can be ordered as part of colorectal cancer screening (multiple options can be selected)

Colonoscopy every 10 years

Flexible sigmoidoscopy every 5 years

CT Colonography every 5 years

Double contrast barium enema every 5 years

Sigmoidoscopy every 10 years plus yearly Annual Fecal immunochemical testing

Annual Fecal DNA testing

Fecal DNA testing every 3 years

Yearly Fecal Immunochemical testing in office

Yearly Fecal immunochemical testing in home

Yearly FOBT at home

Yearly FOBT at office

10. Which of the following CRC screening tests can prevent CRC beside detection

(Multiple options can be selected)

Colonoscopy every 10 years

Flexible sigmoidoscopy every 5 years

CT Colonography every 5 years

Double contrast barium enema every 5 years

Sigmoidoscopy every 10 years

Annual Fecal DNA testing

Fecal DNA testing every 3 years

Yearly Fecal Immunochemical testing in office

Yearly Fecal immunochemical testing in home

Yearly FOBT at home

Yearly FOBT at office

11. Which of the following CRC screening tests only detect CRC without prevention

(Multiple options can be selected)

Colonoscopy every 10 years

Flexible sigmoidoscopy every 5 years

CT Colonography every 5 years

Double contrast barium enema every 5 years

Sigmoidoscopy every 10 years

Annual Fecal DNA testing

Fecal DNA testing every 3 years

Yearly Fecal Immunochemical testing in office

Yearly Fecal immunochemical testing in home

Yearly FOBT at home

Yearly FOBT at office

12. Which of the following are considered the alternative methods of CRC screening

(Multiple options can be selected)

Colonoscopy every 10 years

Flexible sigmoidoscopy every 5 to 10 years

CT Colonography every 5 years

Double contrast barium enema every 5 years

Annual Fecal DNA testing

Fecal DNA testing every 3 years

Yearly Fecal Immunochemical testing in office

Yearly Fecal immunochemical testing in home

Yearly FOBT at home

Yearly FOBT at office

13. Colonoscopy should be done next if which of the following tests are positive

(Multiple options can be selected)

Flexible sigmoidoscopy

CT Colonography

Double contrast barium enema

Fecal DNA testing

Fecal Immunochemical testing

FOBT

14. Patients with small rectal hyperplastic polyps on colonoscopy should undergo follow up colonoscopy in

10 Years

5 Years

3 Years

1 Year

15. Patients with 1 or 2 small (less than 1 cm) tubular adenomas with low-grade dysplasia should undergo follow up colonoscopy

5 Years

10 Years

1 Year

3 Years

3 to 5 Years

16. Patients with 3 to 10 adenomas, or a large (at least 1 cm) adenoma, or any adenomas with high-grade dysplasia or villous features, the follow up colonoscopy

should be done at

3 Years

5 Years

10 Years

1 Year

17. Patients with more than 10 adenomas on a single exam, the follow up colonoscopy

should be done at

1 to 3 Years

1 Year

10 Years

3 Years

5 Years

18. Patients with sessile adenomas that are removed in pieces, the follow up colonoscopy should be done at

6 Months

2 to 6 Months

1 Year

1 to 3 Years

3 Years

5 Years

19. Familial adenomatous polyposis (FAP) diagnosed by genetic testing, or suspected FAP without genetic testing, which of the following is appropriate screening strategy

Annual Flexible sigmoidoscopy beginning at age 10 years

Annual Colonoscopy at age 25

Annual CT colonography at age 20

Annual FIT at age 12

20. Lynch syndrome (hereditary non-polyposis colon cancer or HNPCC), or at increased risk of Lynch syndrome based on family history without genetic testing, which of the following is appropriate screening strategy

Annual Flexible sigmoidoscopy beginning at age 10 years

Annual Colonoscopy at age 25

Annual CT colonography at age 20

Annual FIT at age 12

21. In a patient diagnosed to have Inflammatory bowel disease (Chronic ulcerative colitis or Crohn’s disease)

which of the following is appropriate screening strategy

Annual Flexible sigmoidoscopy 10 years after diagnosis

Annual Colonoscopy 8 years after diagnosis

Annual CT colonography 5 years after diagnosis

Annual FIT 3 years after diagnosis
