# Supplementary material for: Improving Internal Medicine Residents’ Colorectal Cancer Screening Knowledge Using a Smartphone App: Pilot Study
Source: JMIR Med Educ. 2018 Mar 13;4(1):e10. doi: 10.2196/mededu.9635 (PMC5871737; doi:10.2196/mededu.9635)
Supplement: Multimedia Appendix 2 [file mededu_v4i1e10_app2.pdf]

Number of responders correctly identifying screening in average-risk and with positive family history of colorectal cancer

CORRECT RESPONSE	PRE	POST		PRE	POST		PRE	POST		PRE-TEST				POST-TEST				PRE	POST	
SCREENING EXAM	PGY 1 n=22	PGY 1 n=20	P	PGY 2 n=15	PGY 2 n=11	P	PGY 3 n=13	PGY 3 n=10	P	PGY 1 n=22	PGY 2 n=15	PGY 3 n=13	P	PGY 1 n=20	PGY 2 n=11	PGY3 n=10	P	Total n=50	Total n=41	P
Colonoscopy at 50 (Average)	21 (95.5%)	19 (95.0%)	1	15 (100.0%)	11 (100.0%)	1	12 (92.3%)	10 (100.0%)	1	21 (95.5%)	15 (100.0%)	12 (92.3%)	0.731	19 (95.0%)	11 (100.0%)	10 (100.0%)	1	48 (96.0%)	40 (97.5%)	0.678
Colonoscopy at 45 (African American)	4 (18.2%)	16 (80.0%)	0.00	0 (0.0%)	8 (72.7%)	0.00	0 (0.0%)	5 (50.0%)	0.007	4 (18.2%)	0 (0.0%)	0 (0.0%)	0.112	16 (80.0%)	8 (72.7%)	5 (50.0%)	0.247	4 (8.0%)	29 (72.5%)	0.000
Preventive Tests First	7 (31.2%)	11 (55.0%)	0.212	7 (46.6%)	8 (72.7%)	0.246	6 (46.2%)	8 (80.0%)	0.197	7 (31.2%)	7 (46.6%)	6 (46.2%)	0.613	11 (55.0%)	8 (72.7%)	8 (80.0%)	0.364	20 (40.0%)	27 (65.8%)	0.014
1st Degree Relative with Age > 60, Colonoscopy=40 0r 50	22 (100.0%)	19 (95.0%)	0.476	14 (93.3%)	11 (100.0%)	1	12 (92.3%)	8 (80.0%)	0.560	22 (100.0%)	14 (93.3%)	12 (92.3%)	0.309	19 (95.0%)	11 (100.0%)	8 (80.0%)	0.209	48 (96.0%)	38 (92.7%)	0.654
1st Degree Relative with CRC Age < 60, C=40	21 (95.5%)	19 (95.0%)	1	15 (100.0%)	11 (100.0%)	1	12 (92.3%)	9 (90.0%)	1	21 (95.5%)	15 (100.0%)	12 (92.3%)	0.731	19 (95.0%)	11 (100.0%)	9 (90.0%)	0.732	48 (96.0%)	39 (95.1%)	1
1st Degree Relative with CRC <60, C Every 5 Years	9 (40.9%)	11 (55.0%)	0.361	5 (33.3%)	4 (36.4%)	1	1 (7.7%)	3 (30.0%)	0.281	9 (40.9%)	5 (33.3%)	1 (7.7%)	0.114	11 (55.0%)	4 (36.4%)	3 (30.0%)	0.394	15 (30.0%)	18 (43.9%)	0.170
