# Supplementary material for: Improving Internal Medicine Residents’ Colorectal Cancer Screening Knowledge Using a Smartphone App: Pilot Study
Source: JMIR Med Educ. 2018 Mar 13;4(1):e10. doi: 10.2196/mededu.9635 (PMC5871737; doi:10.2196/mededu.9635)
Supplement: Multimedia Appendix 3 [file mededu_v4i1e10_app3.pdf]

Number of responders correctly identifying the screening tests for CRC

CORRECT RESPONSE	PRE	POST		PRE	POST		PRE	POST		PRE-TEST				POST-TEST				PRE	POST	
SCREENING EXAM	PGY 1 n=22	PGY 1 n=20	P	PGY 2 n=15	PGY 2 n=11	P	PGY 3 n=13	PGY 3 n=10	P	PGY 1 n=22	PGY 2 n=15	PGY 3 n=13	P	PGY 1 n=20	PGY 2 n=11	PGY3 n=10	P	Total n=50	Total n=41	P
Colonoscopy Every 10 Years	22(100.0%)	17 (85.0%)	0.099	14 (93.3%)	11 (100.0%)	1	13(100.0%)	9 (90.0%)	0.435	22 (100.0%)	14 (93.3%)	13 (100.0%)	0.560	17 (85.0%)	11 (100.0%)	9 (90.0%)	0.670	49 (98.0%)	37 (90.2%)	0.171
Flexible Sigmoidoscopy Every 5 Years	18 (81.2%)	13 (65.0%)	0.298	9 (60.0%)	9 (81.2%)	0.395	9 (69.2%)	10 (100.0%)	0.105	18 (81.2%)	9 (60.0%)	9 (69.2%)	0.262	13 (65.0%)	9 (81.2%)	10 (100.0%)	0.065	36 (72.0%)	32 (78.0%)	0.509
CT Colonography Every 5 Years	5 (22.7%)	10 (50.0%)	0.065	4 (26.7%)	7 (63.4%)	0.109	3 (23.0%)	5 (50.0%)	0.221	5 (22.7%)	4 (26.7%)	3 (23.0%)	1	10 (50.0%)	7 (63.4%)	5 (50.0%)	0.780	12 (24.0%)	22 (53.6%)	**0.005**
Double Contrast Barium Enema Every 5 Years	3 (13.7%)	7 (35.0%)	0.152	0 (0.0%)	6 (54.5%)	**0.002**	0 (0.0%)	6 (60.0%)	**0.002**	3 (13.7%)	0 (0.0%)	0 (0.0%)	0.246	7 (35.0%)	6 (54.5%)	6 (60.0%)	0.340	3 (6.0%)	19 (46.3%)	**0.000**
Sigmoidoscopy Every 10 years + FIT Yearly	3 (13.7%)	8 (40.0%)	0.081	3 (20.0%)	6 (54.5%)	0.103	2 (15.4%)	5 (50.0%)	0.169	3 (13.7%)	3 (20.0%)	2 (15.4%)	0.888	8 (40.0%)	6 (54.5%)	5 (50.0%)	0.717	8 (16.0%)	19 (46.3%)	**0.002**
Fecal DNA testing 1-3 Years	8 (36.3%)	15 (75.0%)	**0.016**	6 (40.0%)	5 (45.5%)	1	4 (30.8%)	4 (40.0%)	0.685	8 (36.3%)	6 (40.0%)	4 (30.8%)	0.932	15 (75.0%)	5 (45.5%)	4 (40.0%)	0.102	18 (36.0%)	24 (58.5%)	**0.032**
Yearly FIT (Fecal Immunochemical Testing) at Home	3 (13.7%)	8 (40.0%)	0.081	1 (6.7%)	4 (36.4%)	0.356	1 (7.7%)	4 (40.0%)	0.127	3 (13.7%)	1 (6.7%)	1 (7.7%)	0.851	8 (40.0%)	4 (36.4%)	4 (40.0%)	1	5 (10.0%)	16 (39.0%)	**0.002**
Yearly FOBT at Home	7 (31.8%)	12 (60.0%)	0.067	2 (13.3%)	5 (45.5%)	0.095	6 (46.2%)	4 (40.0%)	1	7 (31.8%)	2 (13.3%)	6 (46.2%)	0.156	12 (60.0%)	5 (45.5%)	4 (40.0%)	0.560	15 (30.0%)	21 (51.2%)	**0.039**
