# Supplementary material for: Improving Internal Medicine Residents’ Colorectal Cancer Screening Knowledge Using a Smartphone App: Pilot Study
Source: JMIR Med Educ. 2018 Mar 13;4(1):e10. doi: 10.2196/mededu.9635 (PMC5871737; doi:10.2196/mededu.9635)
Supplement: Multimedia Appendix 4 [file mededu_v4i1e10_app4.pdf]

Number of responders correctly identifying the prevention methods

CORRECT RESPONSE	PRE	POST		PRE	POST		PRE	POST		PRE-TEST				POST-TEST				PRE	POST	
SCREENING EXAM	PGY 1 n=22	PGY 1 n=20	P	PGY 2 n=15	PGY 2 n=11	P	PGY 3 n=13	PGY 3 n=10	P	PGY 1 n=22	PGY 2 n=15	PGY 3 n=13	P	PGY 1 n=20	PGY 2 n=11	PGY3 n=10	P	Total n=50	Total n=41	P
**Colonoscopy Every 10 Years**	17 (77.2%)	15 (75.0%)	1	14 (93.3%)	10 (90.1%)	1	13(100.0%)	8 (80.0%)	0.178	17 (77.2%)	14 (93.3%)	13 (100.0%)	0.150	15 (75.0%)	10 (90.1%)	8 (80.0%)	0.613	44 (88.0%)	33 (80.55)	0.323
**Flexible Sigmoidoscopy Every 5 Years**	10 (45.5%)	14 (70.0%)	0.108	6 (40.0%)	8 (72.7%)	0.130	5 (38.5%)	7 (70.0)%	0.214	10 (45.5%)	6 (40.0%)	5 (38.5%)	0.937	14 (70.0%)	8 (72.7%)	7 (70.0)%	1	21 (42.0%)	29 (72.5%)	**0.006**
**CT Colonography Every 5 Years**	1 (4.5%)	11 (55.0%)	**0.000**	1 (6.7%)	6 (54.5%)	**0.021**	2 (15.4%)	6 (60.0%)	**0.039**	1 (4.5%)	1 (6.7%)	2 (15.4%)	0.674	11 (55.0%)	6 (54.5%)	6 (60.0%)	1	4 (8.0%)	23 (56.1%)	**0.000**
**DCBE Every 5 Years**	1 (4.5%)	10 (50.0%)	**0.001**	0 (0.0%)	5 (45.5%)	**0.007**	0 (0.0%)	5 (50.0%)	**0.007**	1 (4.5%)	0 (0.0%)	0 (0.0%)	1	10 (50.0%)	5 (45.5%)	5 (50.0%)	1	1 (2.0%)	20 (48.7%)	**0.000**
