# Supplementary material for: Improving Internal Medicine Residents’ Colorectal Cancer Screening Knowledge Using a Smartphone App: Pilot Study
Source: JMIR Med Educ. 2018 Mar 13;4(1):e10. doi: 10.2196/mededu.9635 (PMC5871737; doi:10.2196/mededu.9635)
Supplement: Multimedia Appendix 5 [file mededu_v4i1e10_app5.pdf]

Number of responders correctly identifying the detection methods

CORRECT RESPONSE	PRE	POST		PRE	POST		PRE	POST		PRE-TEST				POST-TEST				PRE	POST	
SCREENING EXAM	PGY 1 n=22	PGY 1 n=20	P	PGY 2 n=15	PGY 2 n=11	P	PGY 3 n=13	PGY 3 n=10	P	PGY 1 n=22	PGY 2 n=15	PGY 3 n=13	P	PGY 1 n=20	PGY 2 n=11	PGY3 n=10	P	Total n=50	Total n=41	P
**FECAL DNA TESTING (1-3 Years)**	11 (50.0 %)	16 (80.0 %)	0.058	8 (53.3 %)	8 (72.7 %)	0.428	6 (46.2 %)	6 (60.0 %)	0.680	11 (50.0 %)	8 (53.3 %)	6 (46.2 %)	1	16 (80.0 %)	8 (72.7 %)	6 (60.0 %)	0.480	25 (50.0 %)	30 (73.2 %)	**0.025**
**Annual FIT at Home**	5 (22.7 %)	9 (45.0 %)	0.192	0 (0.0 %)	8 (72.7 %)	**0.000**	2 (15.4 %)	5 (50.0 %)	0.169	5 (22.7 %)	0 (0.0 %)	2 (15.4 %)	0.107	9 (45.0 %)	8 (72.7 %)	5 (50.0 %)	0.366	7 (14.0 %)	22 (53.6 %)	**0.000**
**Annual FOBT at Home**	10 (45.5 %)	13 (65.0 %)	0.204	4 (26.7 %)	7 (63.4 %)	0.109	3 (23.0 %)	5 (50.0 %)	0.221	10 (45.5 %)	4 (26.7 %)	3 (23.0 %)	0.380	13 (65.0 %)	7 (63.4 %)	5 (50.0 %)	0.710	17 (34.0 %)	25 (60.9 %)	**0.010**
