# Supplementary material for: Improving Internal Medicine Residents’ Colorectal Cancer Screening Knowledge Using a Smartphone App: Pilot Study
Source: JMIR Med Educ. 2018 Mar 13;4(1):e10. doi: 10.2196/mededu.9635 (PMC5871737; doi:10.2196/mededu.9635)
Supplement: Multimedia Appendix 6 [file mededu_v4i1e10_app6.pdf]

Number of Responders Correctly identifying positive tests to be followed by colonoscopy

CORRECT RESPONSE	PRE	POST		PRE	POST		PRE	POST		PRE-TEST				POST-TEST				PRE	POST	
SCREENING EXAM	PGY 1 n=22	PGY 1 n=20	P	PGY 2 n=15	PGY 2 n=11	P	PGY 3 n=13	PGY 3 n=10	P	PGY 1 n=22	PGY 2 n=15	PGY 3 n=13	P	PGY 1 n=20	PGY 2 n=11	PGY3 n=10	P	Total n=50	Total n=41	P
**Flexible Sigmoidoscopy**	18 (81.2%)	18 (90.0%)	0.665	12 (80.0%)	10 (90.1%)	0.614	8 (61.5%)	8 (80.0%)	0.405	18 (81.2%)	12 (80.0%)	8 (61.5%)	0.400	18 (90.0%)	10 (90.1%)	8 (80.0%)	0.693	38 (76.0%)	36 (87.8%)	0.183
**CT Colonography**	10 (45.5%)	12 (60.0%)	0.346	10 (66.7%)	11 (100.0%)	0.053	4 (30.8%)	7 (70.0)%	0.100	10 (45.5%)	10 (66.7%)	4 (30.8%)	0.186	12 (60.0%)	11 (100.0%)	7 (70.0)%	**0.037**	24 (48.0%)	30 (73.2%)	**0.015**
**DCBE**	7 (31.8%)	11 (55.0%)	0.130	7 (46.6%)	8 (72.7%)	0.246	4 (30.8%)	8 (80.0%)	**0.036**	7 (31.8%)	7 (46.6%)	4 (30.8%)	0.651	11 (55.0%)	8 (72.7%)	8 (80.0%)	0.367	18 (36.0%)	27 (65.8%)	**0.005**
**Fecal DNA Testing**	13 (59.1%)	16 (80.0%)	0.190	7 (46.6%)	10 (90.1%)	**0.036**	7 (53.8%)	8 (80.0%)	0.379	13 (59.1%)	7 (46.6%)	7 (53.8%)	0.772	16 (80.0%)	10 (90.1%)	8 (80.0%)	0.756	27 (54.0%)	34 (82.9%)	**0.003**
**FIT**	9 (40.9%)	15 (75.0%)	**0.033**	8 (53.3%)	10 (90.1%)	0.084	6 (46.2%)	8 (80.0%)	0.197	9 (40.9%)	8 (53.3%)	6 (46.2%)	0.772	15 (75.0%)	10 (90.1%)	8 (80.0%)	0.613	23 (46.0%)	33 (80.55)	**0.001**
**FOBT**	20 (90.1%)	19 (95.0%)	1	13 (86.7%)	9 (81.2%)	1	10 (76.9%)	9 (90.0%)	0.604	20 (90.1%)	13 (86.7%)	10 (76.9%)	0.515	19 (95.0%)	9 (81.2%)	9 (90.0%)	0.557	43 (86.0%)	37 (90.2%)	0.748
