# Supplementary material for: Improving Internal Medicine Residents’ Colorectal Cancer Screening Knowledge Using a Smartphone App: Pilot Study
Source: JMIR Med Educ. 2018 Mar 13;4(1):e10. doi: 10.2196/mededu.9635 (PMC5871737; doi:10.2196/mededu.9635)
Supplement: Multimedia Appendix 7 [file mededu_v4i1e10_app7.pdf]

Number of Responders Correctly identifying follow up after colonoscopy findings

CORRECT RESPONSE	PRE	POST		PRE	POST		PRE	POST		PRE-TEST				POST-TEST				PRE	POST	
SCREENING EXAM	PGY 1 n=22	PGY 1 n=20	P	PGY 2 n=15	PGY 2 n=11	P	PGY 3 n=13	PGY 3 n=10	P	PGY 1 n=22	PGY 2 n=15	PGY 3 n=13	P	PGY 1 n=20	PGY 2 n=11	PGY3 n=10	P	Total n=50	Total n=41	P
**10 Years in Small Hyperplastic Rectal Polyps**	9 (40.9%)	13 (65.0%)	0.119	9 (60.0%)	10 (90.1%)	0.178	13(100.0%)	9 (90.0%)	0.435	9 (40.9%)	9 (60.0%)	13 (100.0%)	**0.001**	13 (65.0%)	10 (90.1%)	9 (90.0%)	0.222	31 (62.0%)	32 (78.0%)	0.099
**5 Years in 1 or 2 Small Tubular adenomas**	9 (40.9%)	9 (45.0%)	0.789	9 (60.0%)	8 (72.7%)	0.683	10 (76.9%)	9 (90.0%)	0.604	9 (40.9%)	9 (60.0%)	10 (76.9%)	0.136	9 (45.0%)	8 (72.7%)	9 (90.0%)	**0.042**	28 (56.0%)	26 (63.3%)	0.474
**3 Years in 3 to 10 Adenomas**	10 (45.5%)	10 (50.0%)	0.768	11 (73.3%)	10 (90.1%)	0.356	10 (76.9%)	8 (80.0%)	1	10 (45.5%)	11 (73.3%)	10 (76.9%)	0.112	10 (50.0%)	10 (90.1%)	8 (80.0%)	0.054	31 (62.0%)	28 (68.2%)	0.099
**1 to 3 Years in > 10 Adenomas**	4 (18.2%)	11 (55.0%)	**0.023**	3 (20.0%)	8 (72.7%)	**0.015**	7 (53.8%)	5 (50.0%)	1	4 (18.2%)	3 (20.0%)	7 (53.8%)	0.073	11 (55.0%)	8 (72.7%)	5 (50.0%)	0.555	14 (28.0%)	24 (58.5%)	**0.003**
**2 to 6 Months in Sessile Adenomas**	4 (18.2%)	10 (50.0%)	**0.049**	3 (20.0%)	7 (63.4%)	**0.043**	4 (30.8%)	6 (60.0%)	0.223	4 (18.2%)	3 (20.0%)	4 (30.8%)	0.686	10 (50.0%)	7 (63.4%)	6 (60.0%)	0.778	11(22.02%)	23 (56.1%)	**0.001**
