# Supplementary material for: Improving Internal Medicine Residents’ Colorectal Cancer Screening Knowledge Using a Smartphone App: Pilot Study
Source: JMIR Med Educ. 2018 Mar 13;4(1):e10. doi: 10.2196/mededu.9635 (PMC5871737; doi:10.2196/mededu.9635)
Supplement: Multimedia Appendix 8 [file mededu_v4i1e10_app8.pdf]

Number of Responders Correctly identifying surveillance for colorectal cancer in various diseases

CORRECT RESPONSE	PRE	POST		PRE	POST		PRE	POST		PRE-TEST				POST-TEST				PRE	POST	
SCREENING EXAM	PGY 1 n=22	PGY 1 n=20	P	PGY 2 n=15	PGY 2 n=11	P	PGY 3 n=13	PGY 3 n=10	P	PGY 1 n=22	PGY 2 n=15	PGY 3 n=13	P	PGY 1 n=20	PGY 2 n=11	PGY3 n=10	P	Total n=50	Total n=41	P
**Annual Flexible sigmoidoscopy beginning at age 10 years in FAP**	8 (36.3 %)	11 (55.0 %)	0.226	2 (13.3 %)	8 (72.7 %)	**0.004**	3 (23.0 %)	6 (60.0 %)	0.102	8 (36.3 %)	2 (13.3 %)	3 (23.0 %)	0.272	11 (55.0 %)	8 (72.7 %)	6 (60.0 %)	0.648	13 (26.0 %)	25 (60.9 %)	**0.001**
**Annual Colonoscopy in Lynch Syndrome at 25 Years**	16 (72.7 %)	14 (70.0 %)	0.845	12 (80.0 %)	10 (90.1 %)	0.614	9 (69.2 %)	8 (80.0 %)	0.660	16 (72.7 %)	12 (80.0 %)	9 (69.2 %)	0.846	14 (70.0 %)	10 (90.1 %)	8 (80.0 %)	0.440	37 (74.0 %)	32 (78.0 %)	0.654
**Annual Colonoscopy 8 years after diagnosis of IBD**	20 (90.1 %)	17 (85.0 %)	0.656	13 (86.7 %)	10 (90.1 %)	1	10 (76.9 %)	8 (80.0 %)	1	20 (90.1 %)	13 (86.7 %)	10 (76.9 %)	0.515	17 (85.0 %)	10 (90.1 %)	8 (80.0 %)	0.861	43 (86.0 %)	35 (85.4 %)	0.932
